# *Cryptosporidium* species and subtypes in Norway: predominance of *C. parvum* and emergence of *C. mortiferum*

**DOI:** 10.1080/22221751.2024.2412624

**Published:** 2024-10-03

**Authors:** Jahid Hasan Tipu, Audun Sivertsen, Jan-Egil Afset, Lars Sandven, Hanne Brekke, Hilde Marie Lund, Linnea Sofie Elburg, Peter Gaustad, Tore Lier, Liv Reidun Tverelv, Øystein Haarklau Johansen, Lucy J. Robertson, Kurt Hanevik

**Affiliations:** a Department of Global Public Health and Primary Care, Faculty of Medicine, University of Bergen, Bergen, Norway; b Department of Clinical Science, Faculty of Medicine, University of Bergen, Bergen, Norway; c Department of Microbiology, Haukeland University Hospital, Bergen, Norway; d Department of Clinical and Molecular Medicine, Faculty of Medicine and Health Sciences, Norwegian University of Science and Technology, Trondheim, Norway; e Department of Medical Microbiology, St. Olavs Hospital, Trondheim University Hospital, Trondheim, Norway; f Department of Internal Medicine, Førde Central Hospital, Førde, Norway; g Department of Medical Microbiology, Oslo University Hospital, Oslo, Norway; h Norwegian Institute of Public Health, Oslo, Norway; i Fürst Medical Laboratory, Oslo, Norway; j Department of Microbiology, Institute of Clinical Medicine, University of Oslo, Oslo, Norway; k Department of Microbiology and Infection Control, University Hospital of North Norway, Tromsø, Norway; l Department of Microbiology, Southern Health and Social Care Trust, Craigavon, Northern Ireland; m Department of Paraclinical Sciences, Faculty of Veterinary Medicine, Norwegian University of Life Sciences, Ås, Norway; n National Centre for Tropical Infectious Diseases, Haukeland University Hospital, Bergen, Norway

**Keywords:** Cryptosporidiosis, molecular epidemiology, imported, seasonality, regionality, nested PCR

## Abstract

PCR-based diagnostics has revealed the previously largely unknown *Cryptosporidium* transmission and infections in high-income countries. This study aimed to determine domestic and imported subtypes of *Cryptosporidium* species in Norway, evaluate their demographic distribution, and identify potential small outbreaks. *Cryptosporidium*-positive human faecal samples were obtained from six medical microbiology laboratories between February 2022 and January 2024, together with 22 *Cryptosporidium*-positive animal samples. Species and subtypes were identified by sequencing PCR products from gp60 and SSU rRNA genes. Most cryptosporidiosis cases occurred during late summer/early autumn, primarily in children and young adults. Of 550 human samples, 359 were successfully characterized molecularly (65%), revealing infection with 10 different *Cryptosporidium* species. *C. parvum* occurred in 245 (68%) human isolates with IIa and IId being major allele families, with distinct regional distribution patterns of common subtypes. A kindergarten outbreak with 5 cases was due to *C. parvum* IIaA14G1R1. *C. mortiferum* was identified in 33 (9.2%) human cases of which 24 were known to be of domestic origin, making it the second most common species in human autochthonous cases in Norway. All *C. mortiferum* isolates were of the same genotype; XIVaA20G2T1, including 13 cases from a suspected small outbreak in Trøndelag. *C. hominis* occurred in 68 typed cases (19%), but mostly in infections acquired abroad, with allele families Ib and If occurring most often. In conclusion, this study of recent *Cryptosporidium* spp. and subtypes in Norway, highlights the predominance of *C. parvum* and the emergence of *C. mortiferum* among autochthonous cases.

## Introduction

Cryptosporidiosis is a parasitic disease responsible for millions of cases of diarrhoea annually, leading to a loss of 12.9 million disability-adjusted life-years (DALYs) and approximately 48,000 deaths globally [1]. Notably, worldwide it is the fifth leading cause of diarrhoeal mortality in children <5 years and is associated with long-term sequelae such as malnutrition and stunted growth [1].

Currently, 44 species of *Cryptosporidium* have been identified, but only a few usually infect humans [2]. *Cryptosporidium hominis* and *C. parvum* are the two predominant species in humans, accounting for approximately 90% of cryptosporidiosis cases globally [2,3]. Furthermore, genetic characterization has revealed more than 120 distinct subtypes (genetically distinct subgroups within a species) among different *Cryptosporidium* spp. [2]. Transmission is primarily through the faecal-oral route, either hand-to-mouth or by ingestion of food and water contaminated with *Cryptosporidium* oocysts [4,5]. Although *Cryptosporidium* infection is common in settings with poor water and sanitation infrastructure, cryptosporidiosis is not uncommon in high-income countries, and several extensive outbreaks have been reported [6–8].

Although cryptosporidiosis is a notifiable disease in most EU and EEA countries, it is still underdiagnosed and underreported [9]. In Norway, reporting of cryptosporidiosis was not mandatory until 2012 [10]. *Cryptosporidium* is an emerging pathogen in Norway, with the annual reported incidence of human infections increasing from 4 cases in 2012 to 539 cases in 2023 [11]. The increase is likely related to increased testing of diarrhoeal cases for *Cryptosporidium* due to the implementation of multi-pathogen multiplex PCR for diagnostics.

Genotyping can offer insights into the local and national epidemiology of the pathogen, inform authorities about transmission patterns, and support efforts to detect and investigate outbreaks. There are hardly any data regarding *Cryptosporidium* spp. circulating in the human population of Norway and their subtypes.

To address this knowledge gap, the primary aim of this study was to identify the circulating species and subtypes of *Cryptosporidium* spp. in Norway. Secondarily, we evaluated the geographical and demographic distribution of *Cryptosporidium* subtypes, associations with seasons, and evaluated the data for detection of potential small outbreaks.

## Materials and methods

### Study period, study area, and sample size

The study was conducted over a period of 2 years, from February 2022 to January 2024. Samples were obtained from six routine medical microbiology laboratories and one veterinary diagnostic laboratory in Norway. The medical microbiology laboratories are located in all four health regions of Norway (South-Eastern, Western, Central, and Northern Norway). A total of 550 total nucleic acid (TNA) eluates were collected from human faecal samples confirmed positive for *Cryptosporidium* by diagnostic PCR at the participating laboratories during the study period. Additionally, we included a total of 22 *Cryptosporidium-*positive samples from animals, diagnosed by immunofluorescent antibody test (IFAT) from the routine veterinary parasitology diagnostic service at NMBU. The age, sex, sampling date, diagnostic real-time PCR Ct value, and patient county were recorded for each reported case. Moreover, data reported from the treating physicians to the Norwegian Surveillance System for Communicable Diseases (MSIS) were collected to determine the origin of infection.

### Extraction of DNA, molecular detection, and subtyping of *Cryptosporidium* spp.

DNA was extracted from animal faecal samples using QIAamp PowerFecal Pro DNA Kits (QIAGEN, Germany) according to the manufacturer's instructions. To determine the species and subtypes of *Cryptosporidium* for all samples, we performed nested PCRs targeting the 60-kDa glycoprotein (gp60) and small subunit ribosomal RNA (SSU rRNA) gene. Initially, we subjected all samples to a gp60 nested PCR, and samples that were negative for gp60 were subsequently subjected to a nested PCR targeting the SSU rRNA (Figure S1).

For the gp60 nested PCR, previously published AL3531 and AL3535 primers were used in the primary reaction, while AL3532 and AL3534 were used in the secondary reaction [12]. For the SSU rRNA nested PCR, we employed 18S1F and 18S1R in the primary PCR reaction, while primers 18S2F and 18S2R were utilized in the secondary reaction [13]. To determine the subtypes of *C. mortiferum* cases, we followed the Guo et al. 2015 protocol, employing Cgp60F1 and Cgp60R1 primers in the primary reaction, followed by Cgp60F2 and Cgp60R2 in the secondary reaction [14].

Secondary PCR products were separated by 1.5% agarose gel electrophoresis. We used ExoSAP-IT Express reagent (ThermoFisher Scientific, USA) to purify the amplified PCR products. For samples with additional non-specific bands, we utilized Wizard SV Gel and PCR Clean-Up System (Promega, USA) to purify the amplicons. Purified amplicons were sent for Sanger sequencing at Genewiz, Germany, and sequenced in forward and reverse directions.

### Data analysis

We utilized Geneious Prime® 2024.0.5 to obtain consensus sequences and for phylogenetic analysis. To determine SSU species/genotype and gp60 allele families and subtypes, raw gp60, and SSU rRNA chromatograms were analyzed directly with the free software tool CryptoGenotyper [15]. We examined the chromatograms in both directions for double peaks suspicious of mixed infections to validate the sequences. In some cases (when there was uncertainty about the species or subtypes), we used the consensus sequence to determine the species using BLAST and manual counting of trinucleotide repeats to determine the subtypes as described by Xiao, 2010 [16]. Additionally, we reran the nested PCR and amplicons were resequenced when sequence quality was suboptimal. Consensus sequences were aligned with reference sequences obtained from the NCBI nucleotide database to ascertain their species and allele families (supplementary figure S5). To analyze the demographic data and for genotype mapping, we used R (v4.4.0; R Core Team 2021) with packages tidyverse, ggplot2 and sf, and Microsoft Excel® (version 2403). Associations between age group and different subtypes were determined using Chi-square test contingency table analysis, with a *p*-value <0.05 as a cut-off for significance.

## Results

Of 624 *Cryptosporidium*-positive human samples from the six medical microbiology participating laboratories during this period, 550 TNA eluates were stored and available for genotyping in this study (Table S1). The median age of the patients from whom positive samples were obtained was 33.5 years, ranging from <1 to 90 years. There was a slightly higher proportion of females (58.5%). Genotyping success rates ranged from 41.7% to 86.4% according to submitting medical microbiology laboratory (Table S1) and was independent of age and sex of the patients (data not shown). Genotyping was successful in 18 of 22 (81.8%) animal faecal samples (Table S1).

### *Cryptosporidium* spp. in humans and animals

Species identification was successful for 359 of 550 human samples (65.3%), revealing 10 different *Cryptosporidium* species. The predominant species was *C. parvum,* accounting for 68.2% (n = 245) of the total, followed by 18.9% *C. hominis* (n = 68), 9.2% *C. mortiferum* (n = 33), and a small number of other species; *C. ubiquitum* (n = 3), *C. felis* (n = 2), *C. canis* (n = 2), *C. erinacei* (n = 2), *C. ditrichi* (n = 2), *C. meleagridis* (n = 1), and *C. tyzzeri* (n = 1).

Out of 18 successfully sequenced isolates from animal faecal samples, 83.3% (n = 15) were identified as *C. parvum,* comprising 2 from calves, 10 from cattle, and 3 from dogs. Additionally, one infection with *C. bovis* from cattle, one with *C. hominis* from sheep, and one with *C. xiaoi* from sheep were identified.

### Demographic and seasonal distribution of *Cryptosporidium* infection

**Figure 1.**
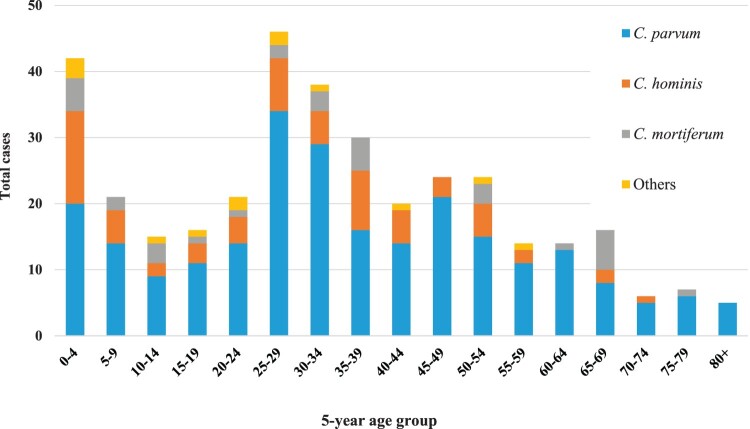
Distribution of *Cryptosporidium* spp. in 359 successfully sequenced human cryptosporidiosis cases in Norway from February 2022 to January 2024 by 5-year age groups.

We categorized the human cryptosporidiosis cases into 5-year age intervals. The bimodal distribution showed that young adults (25–29 years) and children (0–4 years) were more frequently infected (Figure 1). *C. parvum* was the most commonly identified species in all age groups and there seemed to be a small spike of *C. mortiferum* in the 65–69 year age group (Figure 1). Additionally, the proportion of *C. hominis* was significantly higher in children 0–4 years old compared to all other age groups combined (*p* = 0.020). Of the most common subtypes found in children 0–4 years old, the *C. hominis* IeA11G3T3 subtype was significantly more frequent (*p* = 0.027) in the likely parent group (25–34 years), when compared to all other age groups above 4 years of age (Figure S2).

**Figure 2.**
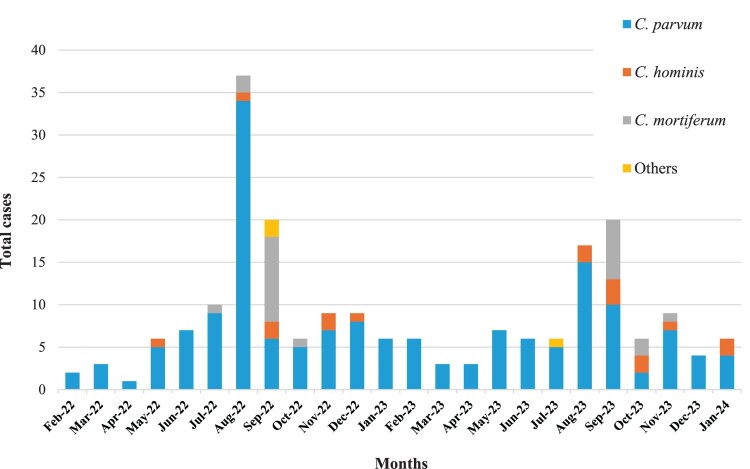
Monthly distribution of *Cryptosporidium* spp. in 209 successfully sequenced human domestic cryptosporidiosis cases in Norway from February 2022 to January 2024.

Most of the domestic cases occurred in late summer and early autumn (August and September) and were lowest in March and April (Figure 2). While *C. parvum* cases peaked in August, *C. mortiferum* cases peaked in September in both 2022 and 2023. In imported cases, there were fewer cases and they increased in number in relation to returns from holidays, especially after the summer 2023 (Figure S3).

### Distribution of *Cryptosporidium* spp. by origin of infection

**Figure 3.**
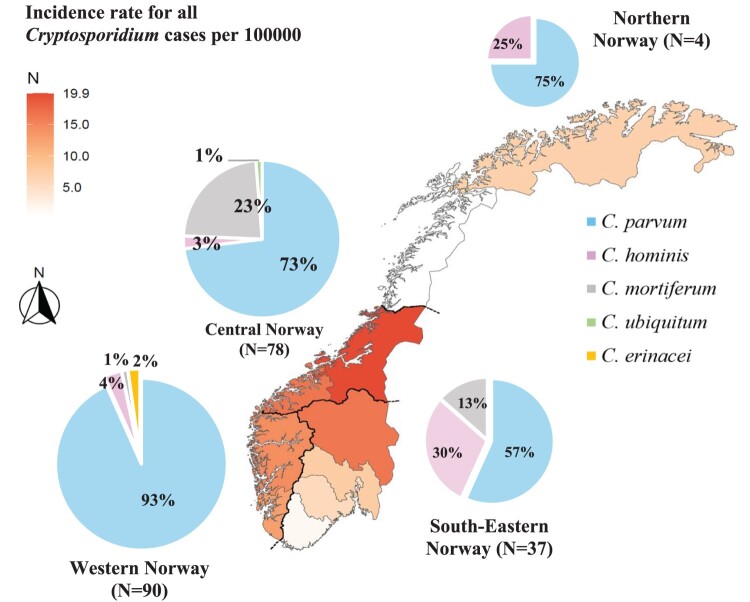
*Cryptosporidium* incidence rate and species distribution of domestic cases. The map indicates the incidence rate per county of all *Cryptosporidium*-positive cases per 100,000 inhabitants in Norway in 2023. Pies show proportions of *Cryptosporidium* spp. in examined cases of domestic origin by the health regions of Norway (separated by black borders) in the study period. Nordland and Agder counties are almost white on the map due to their low incidence rates, which were only 0.4 and 1.6 per 100,000 inhabitants, respectively.

Among the 359 genotyped human samples, data from MSIS showed that 58.2% (n = 209) of the cases were domestically acquired, 23.1% (n = 83) of cases were acquired from abroad (travel-associated), and in18.7% (n = 67) of cases the origin of infection was not reported to MSIS (Table 1). We sub-categorized the domestic infections by Norwegian health regions, which revealed that *C. parvum* was more frequent in Western Norway, *C. hominis* in South-Eastern Norway, and *C. mortiferum* in Central Norway (Figure 3). Furthermore, the infections acquired from abroad (N = 83) were sub-classified according to WHO regions, where all regions except the Western Pacific were represented (Table 1). It is noteworthy that most cases of *C. hominis* infection were not autochthonous, and a substantial portion of these cases were reported to originate in Africa (Table 1).

**Table 1.** Distribution of *Cryptosporidium* spp. in people diagnosed in Norway according to the most likely origin of infection.

Species	Total	Norway	WHO regions						Unknown
			Other European regions	African regions	South-East Asian regions	American regions	Eastern Mediterranean regions	Western Pacific regions	
*C. parvum*	245	165	27	2	2	2	2	0	45
*C. hominis*	68	17	14	21	0	0	5	0	11
*C. mortiferum*	33	24	1	0	0	0	0	0	8
*C. ubiquitum*	3	1	1	0	0	0	0	0	1
*C. felis*	2	0	1	1	0	0	0	0	0
*C. canis*	2	0	0	1	0	0	0	0	1
*C. ditrichi*	2	0	1	0	0	0	0	0	1
*C. erinacei*	2	2	0	0	0	0	0	0	0
*C. meleagridis*	1	0	0	0	1	0	0	0	0
*C. tyzzeri*	1	0	0	1	0	0	0	0	0
**Total**	**359**	**209**	**45**	**26**	**3**	**2**	**7**	**0**	**67**

### *C. hominis*, *C. parvum*, and *C. mortiferum* gp60 allele families, and subtypes

We found five distinct gp60 allele families of *C. hominis* and seven of *C. parvum* with a total of 68 distinct subtypes in humans (Table 2). The most common allele families were Ib (26.6%) and If (25%) for *C. hominis* and IIa (63.7%) and IId (31.8%) for *C. parvum*. In the animal samples, only two allele families of *C. parvum* and one family of *C. hominis* were identified, with a total of four distinct subtypes, but the majority (76.9%) were in the IIa family (Table 2).

**Table 2.** The gp60 allele families of *C. hominis* and *C. parvum* and their subtypes identified in humans and animals in this study.

***C. hominis* allele families (N)**	**Subtype (N)**	***C. parvum* IIa allele family (N)**	**Subtype IIa (N)**	***C. parvum* non-IIa allele families (N)**	**Non-IIa subtypes (N)**
**1. Humans**	** **	** **	** **	** **	** **
Ia (3)	IaA22R3 (1)	IIa (156)	**IIaA10G1R2 (36)**	IIc (4)	IIcA5G3a (2)
** **	IaA25R3 (1)	** **	**IIaA14G1R1 (26)**	** **	IIcA4G3a (1)
** **	IaA26R3 (1)	** **	IIaA18G1R1 (24)	** **	IIcA5G3j (1)
Ib (17)	**IbA9G3 (14)**	** **	IIaA15G2R1 (16)	IId (78)	**IIdA22G1 (27)**
** **	IbA10G2 (2)	** **	IIaA16G1R1 (12)	** **	IIdA18G1 (8)
** **	IbA13G3 (1)	** **	IIaA17G1R2 (9)	** **	IIdA19G1 (7)
Id (15)	IdA16 (5)	** **	IIaA17G1R1 (5)	** **	IIdA24G1 (7)
** **	IdA14 (2)	** **	IIaA13G2R1 (3)	** **	IIdA26G1 (5)
** **	IdA22 (2)	** **	IIaA13R1 (3)	** **	IIdA16G1 (4)
** **	IdA13 (1)	** **	IIaA17G2R1 (3)	** **	IIdA20G1 (4)
** **	IdA15 (1)	** **	IIaA21G1R1 (3)	** **	IIdA23G1 (4)
** **	IdA15G1 (1)	** **	IIaA15G1R2 (2)	** **	IIdA17G1 (3)
** **	IdA18 (1)	** **	IIaA16G2R1 (2)	** **	IIdA21G1 (3)
** **	IdA21 (1)	** **	IIaA9R1 (1)	** **	IIdA15G1 (2)
** **	IdA25 (1)	** **	IIaA10G3R1 (1)	** **	IIdA25G1 (2)
Ie (13)	**IeA11G3T3 (12)**	** **	IIaA13G1R1 (1)	** **	IIdA13G1 (1)
** **	IeA14G4 (1)	** **	IIaA14R2 (1)	** **	IIdA14 (1)
If (16)	**IfA12G1R5 (14)**	** **	IIaA15G1R1 (1)	IIm (2)	IImA14G1 (1)
	IfA13G1R4 (1)	** **	IIaA16G3R1 (1)	** **	IImA7G1 (1)
	IfA9G1R5 (1)	** **	IIaA18G2R1 (1)	IIq (3)	IIqA23 (2)
		** **	IIaA19G2R1 (1)	** **	IIqA24 (1)
		** **	IIaA19G3R1 (1)	IIt (1)	IItA12R1 (1)
		** **	IIaA19G5R1 (1)	Ixa (1)	IxaA6 (1)
		** **	IIaA20R1 (1)		
		** **	IIaA22G1R1 (1)		
**2. Animals**		** **			
Ib (1)	IbA9G3 (1)	IIa (10)	**IIaA22G1R1 (8)**	IId (3)	IIdA18G1 (3)
			IIaA10G1R2 (2)		

The most frequent subtypes were shown in bold.

**Figure 4.**
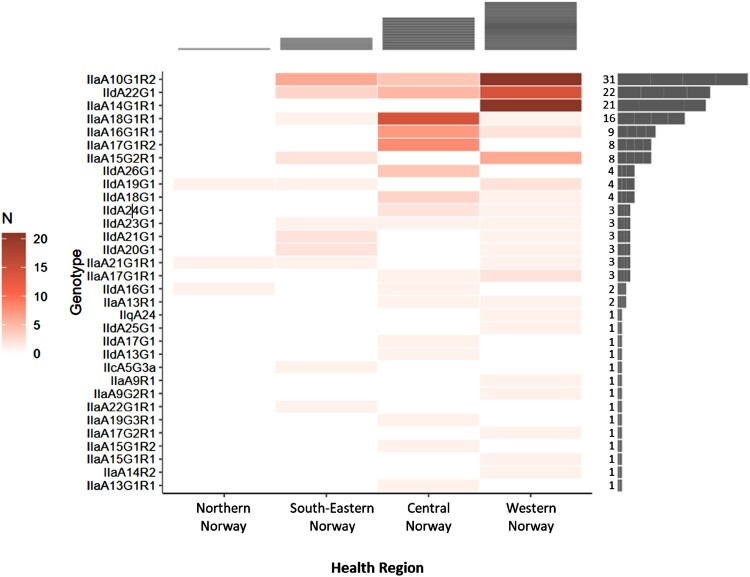
Geographic distribution and frequency of *C. parvum* gp60 subtypes of domestic origin in by health region of Norway during the study period. Each health region is represented as a distinct column, with the number of identified subtypes indicated by coloured bars within the region. The colour intensity of each bar corresponds to the relative frequency of each subtype in that specific health region. Subtypes exhibited different geographic distributions in two health regions (Western and Central) where sufficient samples were available to evaluate them.

We categorized the *C. parvum* subtypes of domestic origin by Norwegian health regions, revealing regional clustering of some subtypes (Figure 4). For instance, IIaA10G1R2, IIaA14G1R1, and IIdA22G1 subtypes were more common in Western Norway. On the other hand, IIaA18G1R1 was more frequent in the health region of Central Norway.

### Potential small outbreaks of cryptosporidiosis

Infection with different *C. parvum* subtypes were generally spread throughout the study period, but a cluster of cases with IIaA14G1R1 subtype occurred during the summer 2022, connected to a kindergarten outbreak with five confirmed cases within 2 weeks in August 2022.

Out of 33 *C. mortiferum* isolates, 28 were successfully subtyped and all were classified as subtype XIVaA20G2T1. Infection with *C. mortiferum* seemed to predominate in elderly people aged 65–69 years. Further investigation of the *C. mortiferum* cases revealed that 13 cases were reported within three weeks from Trøndelag region, suggesting a previously unrecognized *C. mortiferum* XIVaA20G2T1 outbreak in September 2022.

## Discussion

During the study period, we identified a diverse range of *Cryptosporidium* spp. and gp60 allele families and subtypes. *C. parvum* was the predominant species, followed by *C. hominis* and *C. mortiferum*. In addition, we observed some notable trends in species and subtypes in different age groups, seasons, and reported origin of infection.

### Demographic and seasonal distribution

Cryptosporidiosis is recognized as a paediatric disease worldwide, mainly infecting children 6 months to 2 years of age [9]. The present investigation revealed that cryptosporidiosis was common in all age groups in Norway, but that young children and adults 25–29 years of age were more frequently infected. A recent study conducted in Finland also reported similar findings, with the highest occurrence rate in adults of the 25–29 years age group [17]. Being a parent of young children, with a risk of household transmission [18], as well as having a more active lifestyle and travelling, are likely contributing factors to the increased frequency in this group. The age distribution observed in our study, where *C. hominis* predominated in children and *C. parvum* in adults, aligns with patterns reported in the Netherlands [19].

Although this study was not initially designed to evaluate the seasonality of infection, cryptosporidiosis was more commonly diagnosed in late summer and early autumn (August and September). A similar pattern, with more cases during the late summer and early autumn, was reported in a review conducted in European countries [9] and recently from the Swedish surveillance system [20]. In Norway, people are frequently engaged in outdoor activities, including camping, swimming, fishing, hunting, hiking, berry-picking, and mushroom harvesting, during this period of the year, and such activities may increase the likelihood of ingesting *Cryptosporidium* oocysts from contaminated water or from contact with domestic or wild animals or their faeces. Additionally, the observed peaks in human cryptosporidiosis may be linked to the calving season, as the incidence rate documented in this study closely aligns with the geographical distribution of cattle herds in Norway [21]. The calving season could increase the environmental presence of *Cryptosporidium*, thereby such exposure potentially contributes to elevated infection rates.

### C. parvum

*C. parvum* being the most common species found in Norway, agrees with similar studies conducted in Sweden and Finland, which found that *C. parvum* accounted for 79% and 70% of the total human cases, respectively [17,22]. In this study, the most common *C. parvum* gp60 allele families were IIa and IId, with the IIa subtype also reported to be the dominant allele family in previous studies conducted in Sweden, France, Finland, and Denmark [17,22–24].

Somewhat surprisingly, IIaA10G1R2 was the most common subtype of *C. parvum* identified in this study; this subtype has not been identified in Europe. The most common subtype circulating in Europe is IIaA15G2R1 [9], but was only the fourth most common subtype within the IIa family in our study.

The second most common IIa subtype was IIaA14G1R1. This subtype is also common in other European countries [20,22,23,25], and was the likely cause of a small apple-juice-associated outbreak in Norway in 2018 [26]. In our study, we also detected this subtype to be associated with a kindergarten outbreak in Øygarden, in Western Norway.

*C. parvum* IIc subtype is genetically distinct and anthroponotic in nature and found only in humans [16]. The present study identified only four cases of IIc *C. parvum*, including one case with IIcA5G3j subtype of unknown origin, which was recently reported in two cases from Sweden [20,22].

Similar to the IIc allele family, IIm might be restricted to humans, as there have not yet been any cases reported from animals [27]. We found two human cases with this subtype, both of them presumed to have been associated with travel (one to Denmark and one to Eritrea). The IIm family may be uncommon in Nordic countries, as we were unable to find other published reports about it.

IIdA22G1 was the most common *C. parvum* IId subtype identified in this study. IId subtypes were reported in a Swedish study that found three different variants, one of which was associated with a foodborne outbreak [22]. In 2019, there was a small outbreak of cryptosporidiosis in Italy associated with the IIdA25G1 subtype of *C. parvum* [28]. This subtype was also detected in our study with two confirmed cases: one domestic case from Vestland county, while the origin for the other was not known.

### C. hominis

*C. hominis* is considered anthroponotic, with transmission largely occurring between humans [29]. This aligns well with our finding of a higher proportion *C. hominis* in the youngest age group, and a bimodal age distribution. Also, a *C. hominis* genotype frequently found in children was being more frequent in persons of parental age. Similar bimodal distributions were also reported from many other studies, for example in Sweden and USA [30,31].

The two most common gp60 allele families of *C. hominis* identified in this study were Ib and If. Other European studies also reported Ib as the predominant subtype family [20,22,23,32,33], followed by the Ia [22,23] and Ie family [20,32,33].

Our results suggest that subtype IbA9G3 is endemic and dominant in Norway. By contrast, we found only two cases of the IbA10G2 subtype, the most common *C. hominis* subtype previously reported in Europe [9]. The IbA10G2 subtype was associated with the largest waterborne outbreak of cryptosporidiosis in Europe which occurred in 2010–2011 in Sweden, affecting 45,500 individuals, [7,8] and also the largest waterborne outbreak in the world in Milwaukee, USA through drinking water, which affected around 403,000 residents [34].

Subtype IfA12G1R5, is hypothesized to be “hyper-transmissible” due to its emergence and dominance in the USA, Australia, and New Zealand since 2013 [35–37]. In our study, this subtype was the most common subtype of *C. hominis*. A recent study conducted in Spain reported similar findings [38], suggesting a significant shift in the common variants of *C. hominis*. This subtype has also been identified in other European countries including Sweden, Denmark, Germany, Ireland, the Netherlands, and the UK [22,39].

### C. mortiferum

*C. mortiferum* is a species of increasing interest and a newly recognized pathogen in humans. It was previously known as *Cryptosporidium* chipmunk genotype I [40]. Ours is the first report of this species in Norway, where it emerged as the third most commonly identified species. Chipmunks, squirrels, and deer mice act as the primary hosts of this particular species. Analyzing isolates collected from wild animals and infected humans in the USA and Sweden revealed genetic similarities, suggesting a probable zoonotic transmission pathway from wild animals to humans, particularly in rural areas [14]. In Sweden, *C. mortiferum* has been officially recognized as an emerging pathogen due to a notable increase from just 5 cases in 2021 to 59 cases in 2024, making it the second most commonly identified *Cryptosporidium* species [20,22,41]. Similarly, *C. mortiferum* was first identified in Finland in 2023, where it accounted for 21.1% of cases and became the second most commonly reported species [17] and it is of increasing concern as an emerging pathogen, particularly in regions where it has not been previously reported or was unrecognized.

In our study, the high occurrence of *C. mortiferum* subtype XIVaA20G2T1 is notable. This subtype has been reported in two other Nordic countries, Sweden, and Finland [17,20,22,41]. Although, this species has not yet been reported from domestic animals. The findings of only one genotype across Scandinavia suggest the recent introduction of *C. mortiferum* in humans is well-adapted to one or more wildlife hosts, with relatively frequent zoonotic transmission in the late summer months in the Nordic environment.

### Less common *Cryptosporidium* species in humans

In our study, only 3.6% of the human cases were attributed to species other than *C. hominis, C. parvum,* and *C. mortiferum.* Similar findings were reported in Sweden, France, and Spain [20,22,23,33], supporting that other species are uncommon in European countries.

*C. felis*, *C. canis*, and *C. meleagridis* have been reported to be the three most common *Cryptosporidium* spp. identified in humans apart from *C. hominis* and *C. parvum* [2]. We identified only two cases of *C. felis*, however, both were young adults and reported recent travel history; a man who had travelled to Tanzania, and a woman who had travelled to Germany.

### *Cryptosporidium* spp. and subtypes in animals

It has been reported that *C. parvum* is the most common cause of neonatal diarrhoea in dairy farms and is responsible for a significant economic loss [25]. A study conducted in Western Mainland Europe reported that *C. parvum* was the most commonly identified species in farm animals, with 70% of cases in Belgium and 75% in the Netherlands, which is consistent with our findings [42].

In our study, we have only one case of *C. bovis*, which is another common species of *Cryptosporidium* frequently found in cattle and has been reported in different European countries [42]. Additionally, our study reported one case of *C. xiaoi*, which is considered non-zoonotic and has minimal public health impact [43]. However, this species is common in sheep and has previously been reported in Norwegian sheep [43].

Similar to human cases, our study revealed that IIa was the most commonly identified gp60 allele family in animals. The IIaA22G1R1 was the dominant genotype from animal samples in our study, which has also been reported from farm animals in Sweden, Germany, and the Czech Republic [44]. Notably, all four subtypes identified in animals were also reported in humans, suggesting a potential for zoonotic transmission.

### Origin of infection and regionality

In our study, most of the *C. hominis* cases were imported, particularly from the African region. Similar findings were reported in a Swedish study, where 83.7% of *C. hominis* cases were travel-associated [22]. *C. hominis* is reportedly more common in developing countries with poor sanitation [45].

The majority of the *C. mortiferum* cases identified in the present study were domestic, with one case from Finland and the remaining eight cases where the origin of infection was unknown. A recent study conducted in Sweden revealed that all the *C. mortiferum* cases were domestically acquired [22] and in the Finnish study travel information was unknown [17].

Most of the *C. parvum* cases were also of domestic origin and showed a distinct pattern when sorted by health region. A study conducted in Finland also reported some extent of regional variation of *C. parvum* genotype [17]. This regionality may reflect that most *C. parvum* infections are zoonotic.

### Strengths and weaknesses of the study

To the best of the authors’ knowledge, this is the first molecular epidemiological study that provides an overview of the diversity and distribution of *Cryptosporidium* species and subtypes in Norway. Although we were able to retrieve a relatively high number of samples from all health regions within Norway, we may have a sampling bias reflecting the clinical laboratories that participated and were able to collect the most eluates for subtyping. There may be selection bias in species that were detected, as different commercial kit-based qPCR methods were employed at the participating laboratories. Routine qPCR primers are often now known, and some non-*hominis-parvum* species may not be covered by routine PCR at all sites.

We utilized Sanger sequencing to determine the species and subtypes, which may not pick up mixed infections [46]. Therefore, our study may have underestimated the true prevalence of *Cryptosporidium* spp. or subtypes in case of any mixed infections. The limited number of animal samples in this study underscores the need for additional *C. parvum* subtyping from host animals to confirm the zoonotic potential of *C. parvum* and to investigate whether humans and animals share subtypes regionally.

## Conclusion

Our study provides a molecular epidemiological overview of the diversity of *Cryptosporidium* species and subtypes in domestic and imported cases in Norway. *Cryptosporidium* species are widespread in Norway, with zoonotic *C. parvum* being the dominant species both in humans and animals. *C. mortiferum,* an emerging zoonotic pathogen in Scandinavia, was the third most common species, and the second most common species in autochthonous infections, in this study. The majority of the *C. hominis* cases were imported from abroad.

## Supplementary Material

Supplemental Material

## References

[1] Khalil IA, Troeger C, Rao PC, et al. Morbidity, mortality, and long-term consequences associated with diarrhoea from Cryptosporidium infection in children younger than 5 years: a meta-analyses study. Lancet Glob Health. 2018 Jul;6(7):e758–e768. doi:10.1016/S2214-109X(18)30283-3 29903377 PMC6005120

[2] Ryan U, Zahedi A, Feng Y, et al. An update on zoonotic Cryptosporidium species and genotypes in humans. Animals (Basel). 2021;11(11):3307. doi:10.3390/ani11113307 34828043 PMC8614385

[3] Yang X, Guo Y, Xiao L, et al. Molecular epidemiology of human cryptosporidiosis in low- and middle-income countries. Clin Microbiol Rev. 2021 Mar 17;34(2):e00087–19. doi:10.1128/CMR.00087-19 33627442 PMC8549823

[4] Gerace E, Lo Presti VDM, Biondo C. Cryptosporidium infection: epidemiology, pathogenesis, and differential diagnosis. Eur J Microbiol Immunol (Bp). 2019 Dec 25;9(4):119–123. doi:10.1556/1886.2019.00019 31934363 PMC6945992

[5] Tzipori S, Ward H. Cryptosporidiosis: biology, pathogenesis and disease. Microbes Infect. 2002 Aug;4(10):1047–1058. doi:10.1016/S1286-4579(02)01629-5 12191655

[6] Guzman-Herrador B, Carlander A, Ethelberg S, et al. Waterborne outbreaks in the Nordic countries, 1998 to 2012. Euro Surveill. 2015 Jun 18;20(24):21160. doi:10.2807/1560-7917.ES2015.20.24.21160 26111239

[7] Bjelkmar P, Hansen A, Schönning C, et al. Early outbreak detection by linking health advice line calls to water distribution areas retrospectively demonstrated in a large waterborne outbreak of cryptosporidiosis in Sweden. BMC Public Health. 2017 Apr 18;17(1):328. doi:10.1186/s12889-017-4233-8 28420373 PMC5395832

[8] Widerström M, Schönning C, Lilja M, et al. Large outbreak of Cryptosporidium hominis infection transmitted through the public water supply, Sweden. Emerg Infect Dis. 2014 Apr;20(4):581–589. doi:10.3201/eid2004.121415 24655474 PMC3966397

[9] Caccio SM, Chalmers RM. Human cryptosporidiosis in Europe. Clin Microbiol Infect. 2016 Jun;22(6):471–480. doi:10.1016/j.cmi.2016.04.021 27172805

[10] Campbell SM, Pettersen FO, Brekke H, et al. Transition to PCR diagnosis of cryptosporidiosis and giardiasis in the Norwegian healthcare system: could the increase in reported cases be due to higher sensitivity or a change in the testing algorithm? Eur J Clin Microbiol Infect Dis. 2022 May 1;41(5):835–839. doi:10.1007/s10096-022-04426-3 35243573 PMC8893977

[11] MSIS. Cryptosporidiosis cases by place of infection, 2010 - 2022: Meldingssystem for smittsomme sykdommer (MSIS) Statistikk; 2024 [cited 2024 May 7]. Available from: https://allvis.fhi.no/msis/sykdomshendelser?etter=smittested&fordeltPaa=aar&diagnose=309&tidsrom=2010,2022&smittested=10,11,12&diagramtype=stabletSoyle

[12] Alves M, Xiao L, Sulaiman I, et al. Subgenotype analysis of Cryptosporidium isolates from humans, cattle, and zoo ruminants in Portugal. J Clin Microbiol. 2003 Jun;41(6):2744–2747. doi:10.1128/JCM.41.6.2744-2747.2003 12791920 PMC156540

[13] Liu Y, Xiang J, Gao Y, et al. Rapid detection of Cryptosporidium spp. in diarrheic cattle feces by isothermal recombinase polymerase amplification assays. Heliyon. 2023 Oct;9(10):e20794. doi:10.1016/j.heliyon.2023.e20794 37860527 PMC10582492

[14] Guo Y, Cebelinski E, Matusevich C, et al. Subtyping novel zoonotic pathogen Cryptosporidium chipmunk genotype I. J Clin Microbiol. 2015 May;53(5):1648–1654. doi:10.1128/JCM.03436-14 25762767 PMC4400750

[15] Yanta CA, Bessonov K, Robinson G, et al. Cryptogenotyper: a new bioinformatics tool for rapid Cryptosporidium identification. Food Waterborne Parasitol. 2021 June 1;23:e00115. doi:10.1016/j.fawpar.2021.e00115 33748443 PMC7966988

[16] Xiao L. Molecular epidemiology of cryptosporidiosis: an update. Exp Parasitol. 2010 Jan;124(1):80–89. doi:10.1016/j.exppara.2009.03.018 19358845

[17] Häkkänen T, Rimhanen-Finne R, Antikainen J, et al. Molecular characteristics of Cryptosporidium spp. in human cases in five Finnish hospital districts during 2021: first findings of Cryptosporidium mortiferum (Cryptosporidium chipmunk genotype I) in Finland. Int J Parasitol. 2024 Apr;54(5):225–231. doi:10.1016/j.ijpara.2024.01.002 38242277

[18] Johansen ØH, Hanevik K, Thrana F, et al. Symptomatic and asymptomatic secondary transmission of Cryptosporidium parvum following two related outbreaks in schoolchildren. Epidemiol Infect. 2015;143(8):1702–1709. doi:10.1017/S095026881400243X 25268811 PMC4416355

[19] Wielinga PR, de Vries A, van der Goot TH, et al. Molecular epidemiology of Cryptosporidium in humans and cattle in The Netherlands. Int J Parasitol. 2008 Jun;38(7):809–817. doi:10.1016/j.ijpara.2007.10.014 18054936

[20] Bujila I, Troell K, Ögren J, et al. Cryptosporidium species and subtypes identified in human domestic cases through the national microbiological surveillance programme in Sweden from 2018 to 2022. BMC Infect Dis. 2024 Jan 30;24(1):146. doi:10.1186/s12879-024-09049-x 38291399 PMC10826111

[21] Opsal T, Toftaker I, Nødtvedt A, et al. Gastrointestinal nematodes and Fasciola hepatica in Norwegian cattle herds: a questionnaire to investigate farmers’ perceptions and control strategies. Acta Vet Scand. 2021 Dec 4;63(1):52. doi:10.1186/s13028-021-00618-7 34863233 PMC8645080

[22] Lebbad M, Winiecka-Krusnell J, Stensvold CR, et al. High diversity of Cryptosporidium species and subtypes identified in cryptosporidiosis acquired in Sweden and abroad. Pathogens. 2021 Apr 26;10(5):523. doi:10.3390/pathogens10050523 33926039 PMC8147002

[23] Costa D, Razakandrainibe R, Valot S, et al. Epidemiology of cryptosporidiosis in France from 2017 to 2019. Microorganisms. 2020 Sep 4;8(9):1358. doi:10.3390/microorganisms8091358 32899825 PMC7563450

[24] Stensvold CR, Ethelberg S, Hansen L, et al. Cryptosporidium infections in Denmark, 2010-2014. Dan Med J. 2015 May;62(5):A5086. 26050832

[25] Hoque S, Mavrides DE, Pinto P, et al. High occurrence of zoonotic subtypes of Cryptosporidium parvum in Cypriot dairy farms. Microorganisms. 2022 Feb 28;10(3):531. doi:10.3390/microorganisms10030531 35336110 PMC8951114

[26] Robertson LJ, Temesgen TT, Tysnes KR, et al. An apple a day: an outbreak of cryptosporidiosis in Norway associated with self-pressed apple juice. Epidemiol Infect. 2019 Jan;147:e139. doi:10.1017/S0950268819000232 30869057 PMC6518447

[27] Robertson LJ, Johansen Ø H, Kifleyohannes T, et al. Cryptosporidium infections in Africa-How important is zoonotic transmission? A review of the evidence. Front Vet Sci. 2020;7:575881. doi:10.3389/fvets.2020.575881 33195574 PMC7580383

[28] Franceschelli A, Bonadonna L, Cacciò SM, et al. An outbreak of cryptosporidiosis associated with drinking water in north-eastern Italy, August 2019: microbiological and environmental investigations. Euro Surveill. 2022 Sep;27(35):2200038. doi:10.2807/1560-7917.ES.2022.27.35.2200038 36052722 PMC9438396

[29] Leitch GJ, He Q. Cryptosporidiosis-an overview. J Biomed Res. 2012 Jan;25(1):1–16. doi:10.1016/S1674-8301(11)60001-8 22685452 PMC3368497

[30] Insulander M, Silverlås C, Lebbad M, et al. Molecular epidemiology and clinical manifestations of human cryptosporidiosis in Sweden. Epidemiol Infect. 2013 May;141(5):1009–1020. doi:10.1017/S0950268812001665 22877562 PMC9151846

[31] Yoder JS, Beach MJ. Cryptosporidium surveillance and risk factors in the United States. Exp Parasitol. 2010 Jan;124(1):31–39. doi:10.1016/j.exppara.2009.09.020 19786022

[32] de Lucio A, Merino FJ, Martínez-Ruiz R, et al. Molecular genotyping and sub-genotyping of Cryptosporidium spp. isolates from symptomatic individuals attending two major public hospitals in Madrid, Spain. Infect Genet Evol. 2016 Jan;37:49–56. doi:10.1016/j.meegid.2015.10.026 26518912

[33] Segura R, Prim N, Montemayor M, et al. Predominant virulent IbA10G2 subtype of Cryptosporidium hominis in human isolates in Barcelona: a five-year study. PLoS One. 2015;10(3):e0121753. 25816024 10.1371/journal.pone.0121753 PMC4376526

[34] Zhou L, Singh A, Jiang J, et al. Molecular surveillance of Cryptosporidium spp. in raw wastewater in Milwaukee: implications for understanding outbreak occurrence and transmission dynamics. J Clin Microbiol. 2003 Nov;41(11):5254–5257. doi:10.1128/JCM.41.11.5254-5257.2003 14605176 PMC262506

[35] Huang W, Guo Y, Lysen C, et al. Multiple introductions and recombination events underlie the emergence of a hyper-transmissible Cryptosporidium hominis subtype in the USA. Cell Host Microbe. 2023 Jan 11;31(1):112–123.e4. doi:10.1016/j.chom.2022.11.013 36521488 PMC10124589

[36] Braima K, Zahedi A, Oskam C, et al. Retrospective analysis of Cryptosporidium species in Western Australian human populations (2015–2018), and emergence of the C. hominis IfA12G1R5 subtype. Infect Genet Evol. 2019 Sep 1;73:306–313. doi:10.1016/j.meegid.2019.05.018 31146044

[37] Garcia RJ, Pita AB, Velathanthiri N, et al. Species and genotypes causing human cryptosporidiosis in New Zealand. Parasitol Res. 2020 Jul;119(7):2317–2326. doi:10.1007/s00436-020-06729-w 32494897

[38] Peñuelas Martinez M, Carmena D, Guzmán Herrador BR, et al. Marked increase in cryptosporidiosis cases, Spain, 2023. Eurosurveillance. 2024;29(28):2300733. doi:10.2807/1560-7917.ES.2024.29.28.2300733 38994603 PMC11241854

[39] Peake L, Inns T, Jarvis C, et al. Preliminary investigation of a significant national Cryptosporidium exceedance in the United Kingdom, August 2023 and ongoing. Euro Surveill. 2023 Oct;28(43):2300538. doi:10.2807/1560-7917.ES.2023.28.43.2300538 37883039 PMC10604540

[40] Tůmová L, Ježková J, Prediger J, et al. Cryptosporidium mortiferum n. sp. (Apicomplexa: Cryptosporidiidae), the species causing lethal cryptosporidiosis in Eurasian red squirrels (Sciurus vulgaris). Parasit Vectors. 2023 July 15;16(1):235. doi:10.1186/s13071-023-05844-8 37454101 PMC10349434

[41] Bujila I, Troell K, Fischerström K, et al. Cryptosporidium chipmunk genotype I–An emerging cause of human cryptosporidiosis in Sweden. Infect Genet Evol. 2021;92:104895. doi:10.1016/j.meegid.2021.104895 33971308

[42] Pinto P, Ribeiro CA, Hoque S, et al. Cross-border investigations on the prevalence and transmission dynamics of Cryptosporidium species in Dairy Cattle Farms in Western Mainland Europe. Microorganisms. 2021 Nov 20;9(11):2394. doi:10.3390/microorganisms9112394 34835519 PMC8617893

[43] Robertson LJ, Gjerde BK, Furuseth Hansen E. The zoonotic potential of Giardia and Cryptosporidium in Norwegian sheep: a longitudinal investigation of 6 flocks of lambs. Vet Parasitol. 2010 July 15;171(1):140–145. doi:10.1016/j.vetpar.2010.03.014 20381251

[44] Del Coco VF, Córdoba MA, Bilbao G, et al. Cryptosporidium parvum GP60 subtypes in dairy cattle from Buenos Aires, Argentina. Res Vet Sci. 2014 Apr 01;96(2):311–314. doi:10.1016/j.rvsc.2013.12.010 24480390

[45] Xiao L, Feng Y. Molecular epidemiologic tools for waterborne pathogens Cryptosporidium spp. and giardia duodenalis. Food Waterborne Parasitol. 2017 Sep–Dec;8-9:14–32. doi:10.1016/j.fawpar.2017.09.002 32095639 PMC7034008

[46] Zahedi A, Gofton AW, Jian F, et al. Next generation sequencing uncovers within-host differences in the genetic diversity of Cryptosporidium gp60 subtypes. Int J Parasitol. 2017 Sept 1;47(10):601–607. doi:10.1016/j.ijpara.2017.03.003 28495122

